# Ethiopian Health Care Workers’ Insights into and Responses to Intimate Partner Violence in Pregnancy—A Qualitative Study

**DOI:** 10.3390/ijerph17103745

**Published:** 2020-05-25

**Authors:** Bosena Tebeje Gashaw, Berit Schei, Kari Nyheim Solbraekke, Jeanette H. Magnus

**Affiliations:** 1 College of Health Sciences, Jimma University, 1355 Jimma, Ethiopia; 2 Faculty of Medicine, University of Oslo, 0316 Oslo, Norway; j.h.magnus@medisin.uio.no; 3 Department of Public Health and Nursing, Faculty of Medicine and Health Sciences, University of Science and Technology, N-7489 Trondheim, Norway; berit.schei@ntnu.no; 4 Department of Obstetrics and Gynaecology, St. Olav’s Hospital, 7030 Trondheim University Hospital, N-7489 Trondheim, Norway; 5 Institute of Health and Society, University of Oslo, 0318 Oslo, Norway; k.n.solbrakke@medisin.uio.no; 6 Tulane School of Public Health and Tropical Medicine, New Orleans, LA 70112, USA

**Keywords:** pregnancy IPV, health care workers, insights and responses, guidelines and IPV screening tools, training, women’s empowerment, Ethiopia

## Abstract

Violence against women is a global pandemic, with the potential to spread through generations. Intimate partner violence has impacts on women’s sexual, reproductive, and psycho-social health. It can occur during pregnancy and adversely affect the health of both mother and child. Health care workers involved in antenatal care can have a unique role in identifying intimate partner violence and in intervening, preventing, and mitigating its consequences. In this study, the objective was to explore Ethiopian health care workers’ insights of and responses to intimate partner violence in pregnancy. Using an exploratory design, this qualitative study includes ten semi-structured interviews of health care workers representing different antenatal care centers in Jimma, Ethiopia. The content analyses of translated interview notes were conducted with Atlas.ti7 software, (Atlas.ti Scientific Software Development Gmbh, Berlin). The health care workers shared their insights of the consequences of intimate partner violence during pregnancy in addition to their experience with and responses to the victims. There was a limited understanding of the extent of the adverse impacts of intimate partner violence on pregnancy outcomes, as well as the potential long-term health implications. The informants described how they only gave medical treatment for obstetric complications or visible trauma during pregnancy. There was no formal referral to or linkages with other resources. Women’s empowerment and systemic changes in the health care, including training and capacity building, clear guidelines addressing management of intimate partner violence in pregnancy, and inclusion of intimate partner violence screening tools in the Ethiopian antenatal care chart/card, were recommended by the informants. The adverse impacts of intimate partner violence on pregnancy outcomes were poorly understood by the Ethiopian health care workers in this study. They offered limited assistance to the victims and recommended changes in the routine antenatal care (ANC) and health care systems. They identified various policy initiatives focusing on women’s empowerment to reduce intimate partner violence and its complications especially during pregnancy.

## 1. Introduction

Intimate partner violence (IPV) is a pervasive public health problem globally, affecting one in three women [1]. IPV figures are at an epidemic level in the Sub-Saharan Africa [1,2]. It affects more than three in four women in Ethiopia during their lifetime, and more than a third of all pregnant women [3,4,5,6,7]. Furthermore, the IPV prevalence is higher during pregnancy than any obstetric complications routinely screened for during antenatal care (ANC) visits [8,9]. Women exposed to IPV prior to pregnancy, during pregnancy, and/or during the postpartum period are more likely to experience adverse short- and long-term physical, reproductive, and mental health consequences [10,11,12,13,14]. IPV in pregnancy may increase women’s vulnerability to psychological distress and reduce the victim’s coping skills to survive other stressful life events [15]. It also adversely affects the fetus by increasing the risk of intrauterine fetal death, preterm birth, and low birth weight [16,17].

Health care workers (HCWs) are critically significant in advancing the health sectors’ responses to IPV in general and during pregnancy in particular [16]. They can identify women experiencing violence, and provide support and adequate care. Studies show that screening for IPV provides a distinctive opportunity for identification and interventions [18,19,20]. Health care workers, specifically nurses and midwives working at antenatal care (ANC) centers, are in a unique position to address issues related to IPV with pregnant women [17].

However, the personal perceptions and response of HCWs to IPV in pregnancy can serve as a facilitator or as a barrier when integrating IPV prevention, care and supportive services into health care practice [18]. Some HCWs are willing to address IPV in their clinical activities regarding identification of IPV as a preventive health care issue [19], while others do not support including IPV screening tools in their prenatal reports [20,21].

In Ethiopia, 36% women report IPV in pregnancy, and as many as 81% report lifetime physical or emotional IPV experience [7,22]. IPV screening is not included in ANC services in Ethiopia, where 74% of the pregnant women have at least one ANC visit [23]. Thus, ANC clinics provides an ideal ‘window of opportunity’ to address IPV, as this might be the only time most women come into contact with a health care providers.

In order to get a deeper understanding of Ethiopian ANC clinic health care workers insights of and responses to IPV during pregnancy [24], we developed a qualitative study. Considering the rate of IPV in pregnancy, its impact on the health of the pregnant woman and her unborn fetus [25,26], the ANC visit creates an opportunity for HCWs to identify and interact with IPV victims.

## 2. Methods and Participants

### 2.1. Study Design

A qualitative approach using semi-structured interviews was employed to explore the insights and responses of different HCWs from different ANC clinics related to IPV in pregnant women.

### 2.2. Study Setting

The study was conducted from November 2015 to 30 April 2016, in Jimma town, Oromiya regional state, one of the largest cities in Ethiopia. This multi-ethnic town is located 352 km southwest of the capital, Addis Ababa, distinguished by different religions, cultures and languages, with a total population of 177,900 (CSA, 2015).

### 2.3. Participants

Ethical approval was obtained from the Norwegian Regional Ethical Committee, Norway and Jimma university institutional review board, Ethiopia. Permission was provided by the respective administrative health bureaus. We presented the objective of the study to the heads of each institution, who recruited the participants among their staff members working at ANC in Jimma town. Following this, participants were contacted in person to arrange an appointment that was convenient with their working schedule. Participant characteristics included: professional men and women (age 23–51 years); service years ranging 3–32; married and unmarried; of different religions (Table 1).

### 2.4. Data Collection Tools and Procedures

The interview guide was designed to capture the participants’ understanding of the consequences of IPV in pregnancy; individual encounters with and professional responses to pregnant IPV victims; as well as their reflections and recommendations on what could or should be done about IPV in pregnancy. The authors developed the interview guide based on prior experience, expert opinions, and after reviewing relevant literature on the subject. The interview guide was pretested in institutions at other sites with similar characteristics for clarity. We obtained the informants’ voluntary consent prior to the interview after explaining the aim of the study. Before initiating the interviews, measures were taken to make participants relaxed using different techniques (set induction, such as, small talk for a few minutes), which would help them remain conformable during the interview. We also assured participants that their response will be kept confidential and conducted the interviews at their work place, privately.

We prepared for taping the interviews, but as the majority of the informants declined, the primary investigator (first author) conducted face-to-face interviews with two trained note takers having MSc and midwifery backgrounds took comprehensive notes during the sessions. The interviews lasted an hour on average and were conducted in Amharic, as the interviewer, assistants, and interviewees were all able to speak the Amharic language. The richness of the notes were further strengthened by debriefing sessions (discussing the notes) with the informants right after completion of each interview, and text analyses were conducted as planned.

In an effort to minimize bias, participant health care workers were asked to provide their insights and experiences of IPV in pregnancy, not their own personal experiences. We focused on conceptual requirements, and continued data collection until no additional information emerged and we reached saturation [27,28]. The notes were later translated into English by the interview team and checked by others (outside the research team) with English and Amharic proficiency to ensure accuracy. In order to clarify statements, three set of notes were discussed later with the informants, and amended.

### 2.5. Data Processing and Analysis

The consolidated criteria for reporting qualitative research (COREQ) was followed in this study. COREQ is a formal reporting guideline that helps the researchers to comprehensively report crucial features of a qualitative study [29]. The data collection and analysis were conducted concurrently in line with the iterative nature of qualitative methods [30,31].The first author did the data coding and categorizing using the Atlas.ti software. Then the co-authors reviewed and agreed upon the final list of categories and themes.

We used Atlas.ti7 software [32], an efficient device to organize, capture, and analyze data, facilitating sharing between the researchers, as it provide an overview of the findings during the analytical process. This ensured a systematic process of coding, recognizing themes and categories based on previous research on IPV and the emerging issues, while interpreting the content of the textual data, as illustrated in Table 2 in the text. The decision regarding which item belongs to which particular set (i.e., a group, a category, a pattern, a theme) is a matter of judgment for the researcher/s based on preset criteria or purpose [33,34,35,36]. Furthermore, the content analysis, used to analyze our data, involves an interpretation through systematic process of coding while recognizing themes or categories [36,37]. The categories or themes describing the issue are the outcomes of the content analysis and its goal is to provide knowledge and understanding of the issue under study [38,39].

### 2.6. Ethics and Consent

Ethical approval to conduct the study was obtained from Jimma University College of Health Sciences, IRB, Ref No: HRPGC/305/2015 and the Norwegian Regional Ethical Committee (REK), Ref No: 2015/623/REK Nord, in accordance with the Helsinki Declaration of 1975, revised in 2008. Subsequently, permission to initiate the data collection was obtained from the Jimma town health bureau and Jimma University specialized hospital (JUSH) in response to the collaboration letter written from Jimma University College of Health Sciences. Furthermore, informed written and verbal consent from the respondents was obtained before data collection. A list of the participant numbers (code), as well as the signed consent forms were kept locked in a file cabinet, where no one had access to it, and the respondents were fully anonymized to assure the confidentiality of their responses, their voluntary participation and the right to terminate their participation at any time.

Care was also taken that the interviews did not cause ethical or emotional trauma for the participant HCWs or the interview team. The training and preparation prior to the interviews enable the interviewers to discuss various scenarios promoting a professional non-verbal and verbal attitude during the interviews. The HCWs expressed satisfaction with the interview and the discussion on the notes possibly served as a debriefing session, and to enrich our data.

## 3. Results

The themes emerging among the Ethiopian HCWs were: 1.the important adverse IPV outcomes are physical health consequences; 2. recommendation of reconciliation with perpetrator; and 3. health care systems change is imperative if IPV in pregnancy are to be addressed. Each theme is briefly exemplified using direct quotes from the interviews.

### 3.1. Mainly Physical Health Consequences Were Accepted as an Adverse Outcome

Participant HCWs described encountering many pregnant women with varying degrees of IPV-related injuries, including bleeding, abortion, preterm labour or birth, intrauterine death, and stillbirths. However, they do not help them much other than dressing visible trauma and its obstetric complications related to their clinical training. They emphasized that physical violence particularly can adversely affect the health of the mother and fetus, and that they focus their help on it.

“Partner violence during pregnancy especially physical violence is dangerous and may cause the death of woman and fetus, and should be considered as attacking two lives simultaneously”.

(Nurse)

Although the respondents mostly talked about physical impact of IPV, the generic impact IPV has on any aspect of a woman’s life was acknowledged, including that the co-occurrence of pregnancy and IPV may exceed what a pregnant victim can cope with.

“Pregnancy by itself causes different problems and IPV on top of this might be unbearable”.

(Midwife)

The impact of IPV during pregnancy may restrict survivors’ access to every aspect of life and opportunities, including utilization of maternal health services and prenatal care. One participant relates how IPV, specifically a partner’s controlling actions, acts as a barrier for women’s health care utilization.

“Partners who have controlling behavior patrol every step of their wives’ movement and life. They are controlling everything, including her access to money, her movement, and the need for maternal health care service utilization, including antenatal care (ANC) visits”.

(Nurse)

The HCWs also expressed that they do not ask women if they were victims of IPV, as they fear and lack skill in managing the resultant consequences and emotional problems while asking and talking about history of abuse.

“Whenever we meet victims, we only treat visible trauma, stop bleeding and dress open wounds, that is all. Unless they open it up, nobody will ask, as we cannot offer much help if they disclose it”.

(Midwife)

Health care institutions’ linkage with relevant organizations, would improve the responses to victims of partner violence, but there is no such formal arrangement in the studied facilities to refer IPV victim-survivors for further assistances.

“We do not have formal lines to refer intimate partner violence victims; we only treat visible trauma and send them back home”.

(Midwife)

Other reasons given for the limited assistance provided by the HCWs were: the absence of IPV on the items listing of the ANC chart, lack of guidelines on how to manage IPV, lack of skills and knowledge on how to screen, and instructions on how to help, IPV victims. The following statements substantiate their responses:

“Many times I met victims of partner violence with obstetric complications, but I did not help them much, other than clinical management, I myself was even beaten by my husband; I think IPV has nothing to do with ANC, because it is not listed in the ANC card, mainly we focus on whether fetus is injured or not, and check fetal movement andheart rate”.

(Nurse)

“I had a pregnant woman beaten by her husband to death, she started to have vaginal bleeding, and then a dead fetus was expelled. We gave only medical care because there is no guideline to follow, we do not know how to help, and we have no skill for managing it (IPV)”.

(Midwife)

“I met victims many times, especially I do remember a pregnant woman whose husband is heavy Khat (stimulant) user and she told me that he sexually abused and made her to sufferer throughout the night and caused her genital trauma but because it is not indicated in the ANC card, I even do not know how to ask and help out”.

(Nurse)

### 3.2. Reconciliation in IPV

Advising victims to calm down, asking them to bring their husbands to indirectly teach abusers that IPV in pregnancy may affect the fetus (without mentioning what happens (the IPV) between the couple) was strategies expressed by the HCWs. One of the reasons for this was that, because most women had several children, leaving their husband holds many consequences, making advice of reconciliation the only options:

“I usually advice victims to reconcile with their husbands, because children should not be brought up without father”.

(Midwife)

Women’s economic dependency on their husbands, being housewives with nowhere to go if evicted from their house, were also mentioned by the HCWs when justifying advising victims of reconciliation. Comments made by participants are illustrative:

“I met many victims, including pregnant partner violence victim women. As most of them are economically dependent on their husband, I used to advise them to reconcile with their husbands”.

(Nurse)

“I met pregnant woman victim of IPV who developed complication as a consequence, I did nothing, but referred her to specialized hospital, and dead fetus expelled then she got back to her husband”.

(Nurse)

Our study revealed partner violence is perceived to be widespread and not seen as a big deal by many, including police officers. The participants in our study explained that if a woman resorts to police and when the police realize that the perpetrator is her husband, they immediately refer her to neighbors for reconciliation. Additionally, when the partner finds out his wife stepping to police, he would even become more aggressive and fired her from home. In Ethiopia, there is no linkage or system that allows HCWs reporting IPV to police that may assist them to hold perpetrators accountable for their actions, further forcing HCWs to encourage reconciliation over reporting to police. The responses reflect the community acknowledgement and attitude towards IPV, possibly related to the prevailing male-dominated traditional values and gender norms. The following participant quote demonstrates how police downplay IPV:

“Survivors should not go to police directly, rather turn to their family, because if they go to police things will get much worse. I think going to police has no importance, even perpetrator may start joking on her and say let the police save you’’.

(Midwife)

In the discussion, we also discovered victim women’s previous experience of police deterring women to resort to police assistance, and forcing HCWs’ to prefer reconciliation.

“I saw specially one woman severely beaten, I’ve never seen such incident in my life…hmmm, and her flesh was visible. We advised her to go to police but she refused, because she went to police previously and police referred her back to reconcile with her husband”.

(Midwife)

### 3.3. Changes in the Health Care Systems

The participating HCWs highlighted that IPV so far had not been given any priority by Ethiopian government bodies or the community. In order to prevent IPV in pregnancy and its threats to pregnancy outcomes, training and capacity building, developing and use of IPV intervention guidelines, and women’s empowerment were emphasized by the HCWs.

Participants acknowledged the need to train HCWs, including health extension workers working at the grassroots level in Ethiopia, on how to take care of pregnant women experiencing IPV.

“We need to be knowledgeable and teach the public about IPV and its consequences on pregnancy like we do on birth preparedness and complication readiness”.

(Nurse)

The need to develop and use guidelines to manage victimized pregnant women, along with clear referral linkage with other resource centers help HCWs to adequately deal with IPV issues when presented in the ANC setting was also stressed by the participants. HCWs claimed the absence of IPV screening tools in the lists of ANC card as one of the reasons for not asking women about their victim status and considering it as part of their responsibilities.

“We need to have clear guidelines, and IPV screening tools should be included in the ANC and family planning cards so that we can assist victim survivors”.

(Nurse)

The importance of raising public awareness about the harmful effects of IPV on pregnancy, the current legislation and women’s rights related to IPV were highlighted. More specifically, the HCWs emphasized that they need to have training on women’s rights related to IPV in order to be able to assist, to advise victims on what to do and where to go when abused.

“Even we do not know about women’s rights properly, but if we had enough knowledge about the issue, we could teach others”.

(Nurse)

Empowering women through education, employment, and income-generating activities was also emphasized by the participants to bring a sustainable national solution towards the prevention of IPV. Participants particularly underscored the prevention and liberation of women from IPV requires income-generating activities for women. They strongly recommended this, as most women in Ethiopia are housewives and need to have their own earnings. One participant voiced:

“I would suggest a sustainable solution including short-term training especially for housewives, in order to generate their own money”.

(Nurse)

## 4. Discussion

This is one of few qualitative studies in East Africa addressing HCWs perspectives on IPV in pregnancy [40,41]. The participant HCWs expressed a fear that IPV during pregnancy could discourage victims to visit ANC centers and that it could result in obstetric complications such as bleeding and abortion, which is in concert with international studies [26,42]. They also claimed that it is mainly physical violence that gives adverse outcomes on pregnancy and gave their recommendations on what could be done related to IPV during pregnancy and its adversaries.

The HCWs in our study highlighted mainly physical adverse obstetrical outcomes on pregnancy (bleeding and abortion), illustrating a knowledge gap related to the impacts of IPV on pregnancy in general and the impacts of sexual and psychological IPV on pregnancy outcomes in particular. This is supported by prior studies which noted that HCWs are more likely to focus on physical abuse to respond to IPV and/or during prenatal care visits [43,44].

In this Ethiopian context, such response is not unexpected. There is a common proverb in Ethiopian communities, “an insult never tear cloths” which support and illustrate the importance of the physical harm of IPV. However, many studies illustrate the ongoing effect of psychological violence (emotional torture, mental stress, or living under terror) can be as unbearable as physical brutality, and consequently affecting pregnancy outcomes [45,46]. Similarly, not only physical IPV, but also psychological, sexual IPV in pregnancy adversely affect maternal and fetal well-being. For instance, women who are psychologically abused can be at a greater risk of postnatal depression, are more likely to think about harming themselves and their infants [46,47], while emotional IPV is linked with preeclampsia [26,48] and hypertension [26]. In line with the reflections of the HCWs in our study, prior studies have documented the effect IPV has on maternal health service utilization [22,49,50], illustrating that the impacts of IPV in pregnancy is multifaceted [42,51].

### 4.1. The Role of HCWs in Addressing IPV in Pregnancy

HCWs can help address IPV during pregnancy both through screening, provider-based counseling and referral to legal or social services [52]. In the ANC setting, they have a unique opportunity to identify women experiencing IPV, assess feto–maternal well-being, provide ongoing support [53,54], and prevent complications. The 2013 clinical guidelines developed by WHO, recommend that health systems responses to violence against women should be integrated within clinical care at all levels [53]. Contrary to this recommendation, most health sectors have been rather slow to integrate violence against women into their routine practices [53,55,56,57]. The Ethiopian health system is no exception, and is ill-equipped to deal with IPV, as emphasized by our informants.

Most women [58,59], are reluctant to disclose their experiences of IPV because of the traditional gender attitudes and role perception, and possibly because of fear of further violence or other consequences. As evidenced by our study, very few pregnant victims open up to health care professionals after being brutally hurt. The HCWs participating in our study, shared feelings of frustration and powerlessness to deal with abused pregnant women due to their limited knowledge, responsibility and ability to provide any support, consequently limiting their role to obstetric and medical care for those with visible trauma. The HCWs also feared that asking and talking about abuse with the victims may cause unpredictable and harmful results, as it may open a ‘Pandora’s box’, for which they had neither the skills nor the time to deal with. This is in line with an earlier study in Norway that noted the midwives’ “fear of knowing” how to deal with a positive answer was one of the barriers to asking pregnant women about their experiences of violence [60].

The participants in our study also stated how they did not feel mandated to help the IPV victims, as it was not clearly indicated within the domain of health care. This is consistent with other studies illustrating how many HCWs feel poorly prepared to ask questions about domestic violence or to make appropriate referrals if abuse was disclosed [61,62]. Studies also demonstrated that most HCWs never or seldom ask about abuse when a woman presents with injuries [63]. Another study found that midwives perceived IPV as a non-clinical, social, and domestic problem that does not call for their attention [40]. Likewise, studies elsewhere [64,65], and a recent study in Australia showed that community health care professionals’ barriers to screening pregnant women for violence originated from a lack of recognizing it as a part of their role [66]. This was enforced by lack of domestic violence screening policies, lack of time, lack of resources, safety issues, and lack of confidence in undertaking the screening and referral [66]. Contrary to this, a recent study in South Africa demonstrated that HCWs were supportive to addressing IPV in the health care setting, including identification of and responding to IPV in the antenatal care given appropriate training and referral system [19]. Another study in Serbia reported HCWs as willing to help victims of IPV, but that they did not know how [18], demonstrating the gap between HCWs’ willingness to address IPV and the educational and training resources needed.

### 4.2. Reconciliation in IPV during Pregnancy

The participants in our study highlighted that, although the IPV was brutal, the pregnant women might rarely reveal their victim status to the HCWs. Apart from other reasons, the reconciliation recommendations and actions of the nurses and midwives in our study might be affected by the traditional community concepts related to IPV, including the social taboo of discussing family matters with outsiders, the common belief in male superiority, and the complementary female submissiveness [67], indicating how the respondents’ own cultural beliefs may influence their professional attitude or actions regarding IPV [44]. There was a clear lack of concept related to how to manage IPV in an ANC setting. IPV victim management requires addressing women’s psycho-social needs, including respectful and non-judgmental listening; confidential interaction and showing compassion; providing support information; referrals to specialist help; addressing safety concerns, and so forth [68], in addition to good obstetric and medical care.

### 4.3. Systems Change in the Health Care Setting Is Imperative

The participant HCWs highlighted what could be done towards the prevention of IPV in pregnancy and its consequences in Ethiopia, recommending systems change, specifically in health care. The Ethiopian culture and religion do not encourage discussing or disclosing IPV with others [69], nevertheless, the HCWs recommended several issues to be considered. This included public education on the harmful effects of IPV on pregnancy. The government has a responsibility to raise public awareness about the rights of women, as stipulated in the Ethiopian constitution. The HCWs in conjunction with other relevant agencies should play a key role in the prevention of IPV during pregnancy. It is also evidenced that in most high income countries, HCWs perform a primary prevention and/or health promotion role in conjunction with other agencies [42,70,71], where information on IPV are integrated in their routine health education messages and promoting of positive parenting [53]. A systematic evaluation of IPV prevention done in low and middle income countries, highlights the potential effectiveness of structural interventions for IPV prevention [72], signifying that intervention strategies for IPV should vary depending on the socio-cultural, economic and geographic contexts.

#### 4.3.1. Training and Capacity Building

The need for training of the HCWs, including health extension workers (HEWs), and other community health care workers in Ethiopia was emphasized by the participants of our study. Our result is supported by the WHO report which states that community efforts and awareness-raising play a key role in primary prevention [73], which can lead to increased demand for justice, care for the survivors, and punishment for the perpetrators, particularly in settings where resources are limited [74].

Raising the awareness of both the public and the HCWs about legislations on IPV as well as on other women’s rights issues were also highlighted as a need by our study participants. Prior studies demonstrate that training and strengthening of health systems through abuse assessment protocols, capacity building, effective coordination between relevant agencies, and referral networks can enable health care providers to better address violence against women in their practices [17,53].

#### 4.3.2. Develop Intervention Guideline

The participants in our study suggested developing and including IPV screening tools in the Ethiopian ANC chart/card, conducting short-term training for HCWs, creating comprehensive legislation and guidelines on how to manage IPV in general and IPV in pregnancy more specifically, as an imperative to properly assist pregnant victims. In concordance with our findings, other studies recommend the incorporation of an abuse assessment protocol into the routine prenatal clinics as this increases the assessment, identification, and documentation and referral of abused pregnant women [75,76]. Effective intervention in IPV also requires health care services linking with other relevant sectors [69], including with police. But, participants in our study do not support reporting IPV victim survivors to police and they are not mandated to do so, while there are countries (e.g., the US), obligating HCWs’ mandatory reporting of IPV to police [77]. However, such action requires further risk and benefit analysis as it may escalate the rate of IPV and/or may not guarantee to a greater safety and well-being of survivors and HCWs. Thus, a culturally sensitive and carefully designed health systems’ response in addressing IPV in the ANC clinics will be an important step in achieving sustainable impact towards improving maternal and infant health in the country.

#### 4.3.3. Empowering Women

In our study, the participants underlined the need to produce a sustainable solution to prevent IPV in pregnancy and liberate women from IPV by empowering them through education, employment, and income-generating activities. This was especially recommended for housewives, in order for them to generate their own money, which is in concert with other studies stating that empowered women are less likely to experience IPV [78,79]. A recent study also demonstrated that a higher income was associated with lower past-year physical IPV [80]. Among others, possible explanations for a relationship between women’s empowerment and IPV might be that empowered women may have more information on IPV, be more likely to make decision on their own life and thus more likely to seek help from different sources. While disempowered women, including those having financial constraints, have limited ability to access information and health care services [81]. This is reinforced by the societal norms that encourages maintaining the marriage for the sake of children, family, and better social positions or respect [69,82].

Most women in Ethiopia are economically dependent on their husbands [83], and have many children which may force them to stay in abusive relationships. Women’s economic dependency on their partners is also profoundly deterring many women to sending their abusive partner to prison [84]. Although the relationship between women’s empowerment and IPV are inconclusive, it can be argued that, empowering women economically may encourage increased independence and consequently help women to exit from their abusive relationship [85]. In fact, it may not be the sole solution to protect women against IPV, but if combined with education and the transformation of cultural gender norms that negatively affect women, along with providing other services, it may ultimately protect women from IPV [79,86,87]. Additionally, empowering women and improving their status is of societal importance and essential for achieving the national sustainable development goals [88,89].

### 4.4. Limitations and Strengths

To increase the validity of data and reduce bias, we requested verifications and/or corrections to the notes at the end of each session, and used additional interviews when necessary. We accounted for the researchers’ positionality by having two note-takers in addition to the interviewer in the room, in this way minimizing the influence of the unique identities, experiences, and biases that researchers bring to a given study [90]. Similarly, taking into account the nature of qualitative study, subject and participants (i.e., the responses of female participants may be influenced by their own experience of IPV), care was taken that the participants’ focused their responses on the objective of the study.

However, this study has also some limitations. The subject being studied is very sensitive and shaped by social norms and values. Consequently, the participants may not have disclosed the real scenario about the topic or may have provided biased information. As the majority of participants declined to be taped, we did not record the interviews, but we tried to reduce these gaps by running the sessions using the PI moderating the sessions and two note takers (to ensure the participants own responses) who are familiar with the subject matter. The debriefing while discussing the notes with the interviewee after the session was experienced as positive. Summary notes were developed by the main researcher and the assistants immediately after each session.

The gender of the informants was mainly women, and this might put some limitations on the results. On the other hand, as the general profiles of the health care workers who engage in contact with women are women themselves, we find the gender composition to be relevant for the research question of the study.

As in most qualitative studies, our findings cannot be generalized to a larger population. However, in accordance with the quality criteria of transferability, we argue that the knowledge obtained from this study could be of interest for others in similar settings/contexts.

## 5. Conclusions

The analysis of our study highlights that Ethiopian HCWs encounter pregnant IPV victims frequently, however, their understanding of the adverse impacts of IPV on pregnancy and their professional response to IPV are limited. Improving the capacity of HCWs in addressing IPV in pregnancy through training and subsequent capacity building could improve their knowledge base and skills. The HCWs called for interventions aimed at advancing women’s empowerment through education, employment, and income generating activities as means to prevent IPV in pregnancy and its consequences. The informants also recommended that improving and linking the health sector response with other relevant institutions, and incorporating IPV screening tools in the Ethiopian ANC chart/card along with a clear guidelines, could alleviate IPV during pregnancy and its adverse impacts on mother and fetus. Effectiveness studies of cross-sectorial complex interventions, aimed at reducing IPV in Ethiopia, are also warranted.

## Figures and Tables

**Table 1:** Characteristics of the study participants (N = 10).

Participant Characteristics		n
Age	18–35	7
	36–51	3
Sex	Male	1
	Female	9
Marital status	Married	8
	Single	2
Religion	Orthodox	4
	Muslim	3
	Protestant	3
Occupation	Nurse	7
	Midwife	3
Level of Education	Diploma	4
	BSc degree	6
Service years	3–14	7
	15–32	3

**Table 2:** Procedures/steps of data analysis in Atlas Ti, based on the content analysis.

Steps	Descriptions
Familiarizations	All scripts were read to make a general sense out of it and reflect on the overall meaning
Creating file naming	Create file name and save the project (under new hermeneutic unit/analysis project) and add document in the library
Importing files	Import all scripts (#10 Interviews) lined up under ‘P-Docs’
Condensed meaning unites	Open each scripts turn by turn and create meaning unites that gives sense
Coding	Create codes/label (under code manager) Highlight quotations for each scripts, then drug and drop codes for each respective quotations, or create new codes as necessary
Categorizing	Categorize/create family codes (themes) based on similarities
Producing outputs(categories/families)	Open code manager, click on each codes, click on outputs and save each outputs of the codes of categories/families
Data familiarization	For more data familiarization, scripts were read repeatedly alongside field-notes
Result (describing themes and quotations	In the result section—a detail description of each output of categories under the respective themes, based on the content Analysis method (selected for this specific analysis) and include supporting quotations in each respective theme.
Discussion	In the discussion—interpret results and discuss them in light of relevant literatures within the topic.
